# Impact of eligibility for diabetes remission on response to intensive lifestyle intervention in overweight and obese people with type 2 diabetes: The Look AHEAD trial

**DOI:** 10.1111/dom.16650

**Published:** 2025-07-24

**Authors:** Joanna Y. Gong, Agus Salim, Dianna J. Magliano, Jonathan E. Shaw

**Affiliations:** ^1^ Clinical Diabetes and Epidemiology Baker Heart and Diabetes Institute Melbourne Victoria Australia; ^2^ Department of Diabetes and Endocrinology Royal Melbourne Hospital Melbourne Victoria Australia; ^3^ Department of Endocrinology and Diabetes Western Health Melbourne Victoria Australia; ^4^ School of Translational Medicine Monash University Melbourne Victoria Australia; ^5^ Department of Medicine The University of Melbourne Melbourne Victoria Australia; ^6^ School of Population and Global Health The University of Melbourne Melbourne Victoria Australia; ^7^ School of Public Health and Preventive Medicine, Monash University Melbourne Victoria Australia; ^8^ School of Life Sciences La Trobe University Melbourne Victoria Australia

**Keywords:** cardiometabolic, diabetes remission, intensive lifestyle intervention, obesity, overweight, type 2 diabetes

## Abstract

**Aims:**

To achieve remission of type 2 diabetes, resources to support intensive lifestyle intervention (ILI) are directed toward people with diabetes for whom remission is achievable, that is, people with short diabetes duration and lower HbA1c. We aimed to determine if responses to ILI, other than remission, justify this resource allocation.

**Materials and Methods:**

We analysed participants from the Look AHEAD randomised controlled trial who were 20–65 years old with a BMI of 27–45 kg/m^2^. The effect of ILI was assessed after stratification for remission eligibility at baseline (diabetes duration ≤6 years and no insulin therapy). Cox proportional hazards models evaluated the effect of ILI on incident CVD, chronic kidney disease (CKD), and mortality. Linear models with generalised least squares assessed the effect of ILI on weight, HbA1c, systolic blood pressure, LDL, and eGFR.

**Results:**

There were 3105 participants included—60% women, median age 58 years, and median follow‐up time 9 years (IQR 8–10 years). At baseline, 54% were eligible for remission. Remission eligibility status did not modify the effect of ILI on CVD, CKD, or mortality. ILI led to 2.4 kg greater weight loss (−5.53 kg (95% CI −6.02, −5.03) vs. −3.17 kg (95% CI −3.86, −2.49)), and 0.1% greater HbA1c reduction (−0.28% (95% CI −0.33, −0.23) vs. −0.17% (95% CI −0.23, −0.11)) in eligible compared with ineligible people.

**Conclusions:**

The small additional benefit of ILI in people eligible for remission, compared with ineligible people, may not be enough to justify restricting resources for ILI to such eligible people.

## 1 INTRODUCTION

Achieving remission from type 2 diabetes—defined as an HbA1c of <6.5% (48 mmol/mol) and not on glucose‐lowering medication—may reduce medication burden, diabetes complication rates, and mortality.^1^ As such, it is an attractive target for clinicians and people with type 2 diabetes.^2^ Remission can be attained through weight loss, facilitated by lifestyle intervention, pharmacotherapy, or bariatric surgery.

Intensive lifestyle intervention (ILI) has gained favour as a means of achieving remission for some people with type 2 diabetes. Consisting of intensive modifications to diet and physical activity, ILI can achieve remission in a significant proportion of people with type 2 diabetes. In the Look AHEAD trial of ILI, 12% and 10% of participants achieved remission at 1 and 2 years, respectively.^3^ The more intensive DiRECT trial achieved remission in 46% and 36% of participants at 1 and 2 years, respectively.^4^

In view of the benefits and success of inducing type 2 diabetes remission with ILI, programmes delivering ILI to people with type 2 diabetes have been established in a number of countries, including the United Kingdom, Australia, and the United States.^5^, ^6^, ^7^, ^8^ These programmes are often directed toward people with diabetes for whom remission is achievable, that is, shorter diabetes duration, no insulin requirement, lower HbA1c levels. People who are unlikely to achieve type 2 diabetes remission are commonly excluded from these programmes. However, a minority of programmes are provided to a wider population, for example, the Weight Achievement and Intensive Treatment Program (Why WAIT) at the Joslin Diabetes Center, which does not exclude participants based on diabetes duration or glycaemia.^7^

One such programme that restricts ILI to certain participants is the National Health Service (NHS) Type 2 Diabetes Path to Remission Program, modelled on DiRECT.^5^ This is an ILI for people diagnosed with type 2 diabetes within the last 6 years. Compared with no intervention, it costs £1067 per participant to administer the DiRECT intervention, which has been shown to be cost‐effective at 2 years.^9^, ^10^ From September 2020 to December 2022, 7540 people were referred to the NHS Type 2 Diabetes Path to Remission Program; if all participants completed the programme, this would have cost the UK government £8.05 million. Given the large investment into ILI programmes aimed at inducing diabetes remission, we considered whether the current participant selection criteria represent the best use of resources.

The inclusion and exclusion criteria for these restrictive programmes assume that people who are unlikely to achieve type 2 diabetes remission receive less benefit from ILI programmes, as compared with people likely to achieve remission. However, while this may be true for the outcome of diabetes remission, it has not been proven with regard to outcomes such as improvements in weight or glycaemia, or reduction in the risk of complications such as CVD and chronic kidney disease (CKD). Arguably, such outcomes are more important than remission.

The underlying assumption is that the same resources are better spent on inducing remission in people with recent‐onset type 2 diabetes, as compared with treating people with a longer duration of type 2 diabetes. However, this may not be true. For example, it assumes that achieving remission by lowering one individual's HbA1c from 6.6% (49 mmol/mol) to 6.4% (46 mmol/mol) is more worthwhile than lowering another individual's HbA1c from 10.0% (86 mmol/mol) to 8.0% (64 mmol/mol). While it is better to induce diabetes remission than not, the current treatment paradigm and distribution of funds for ILI assume that, since longer diabetes duration increases cardiovascular risk, inducing diabetes remission in a cohort with a lower cardiovascular risk is better than treating diabetes in a cohort at high cardiovascular risk.^11^

Therefore, the primary aim of this analysis was to evaluate whether eligibility for diabetes remission influences the impact of ILI (when compared with diabetes support and education (DSE) only) on cardiometabolic outcomes other than remission. This will address whether the significant financial investment into ILI programmes is being distributed effectively, to people with diabetes who will benefit the most. If ILI cannot be offered to all people with diabetes, we should investigate ways of identifying the people who would benefit the most, using outcomes beyond diabetes remission. This study will also assess whether people with a longer duration of diabetes can attain the same degree of cardiometabolic benefit, even if diabetes remission rates are low.

## 2 MATERIALS AND METHODS

### 2.1 Study design

We performed a post hoc analysis of the Look AHEAD randomised controlled trial. The details of the Look AHEAD trial have previously been described.^12^ Look AHEAD evaluated the effectiveness of ILI on cardiovascular outcomes, as compared with DSE only, in people with type 2 diabetes who were overweight or obese.

From August 2001 to April 2004, a total of 5145 participants were enrolled in Look AHEAD, of whom 4901 were included in the repository dataset available for post‐hoc analyses (sites with many Native American peoples were excluded from the repository dataset). The ILI was terminated in late 2013–2014, after which an observational extension study ran from 2016 to 2020. The current analysis examines the main trial period only; it does not include the extension period.

Diabetes was determined by self‐report verified by medical records, use of glucose‐lowering medications, confirmation from the personal healthcare provider, or laboratory results meeting the American Diabetes Association's 1997 diagnostic criteria (fasting glucose ≥7.0 mmol/L, symptoms of hyperglycaemia with random plasma glucose ≥11.1 mmol/L, or 2‐h plasma glucose ≥11.1 mmol/L after a 75 g oral glucose load).

The ILI implemented reduced caloric intake and increased physical activity to aim for a weight loss of at least 7%. Group and individual counselling sessions occurred weekly for the first 6 months, thereafter occurring with reduced frequency. Specific ILI strategies included a daily calorie goal of 1200–1800 kcal (with <30% from fat and <15% from protein), meal‐replacement products, and at least 175 min of moderate‐intensity physical activity weekly. The control DSE arm received group sessions that covered diet, exercise, and social support, three times annually during years 1–4, then annually for the remainder of the main trial period.

Physical examination data, including weight and systolic blood pressure (SBP) were available annually from years 0 to 11. Laboratory data, including HbA1c, LDL, and estimated glomerular filtration rate (eGFR) were available annually from years 0–4, then at years 6, 8, and 10.

As per the DiRECT trial, people with an HbA1c <6.0% (42 mmol/mol) at baseline were excluded from the current analysis. People in diabetes remission at baseline—defined by an HbA1c of <6.5% (48 mmol/mol) and taking no glucose‐lowering medications—were also excluded. The main analysis was further restricted to participants who were aged 20–65 years old and had a BMI of 27–45 kg/m^2^ at baseline. This matched the age and BMI inclusion criteria of the DiRECT trial,^4^ on which some real‐world ILI programmes are modelled. Additional analyses were conducted without these age and BMI restrictions to ascertain whether differences in effects were due to age and/or BMI restrictions. Henceforth, the population restricted to the DiRECT age and BMI criteria will be referred to as the ‘restricted population’, while the population not restricted to these age and BMI criteria will be referred to as the ‘unrestricted population’.

### 2.2 Statistical methods

The cohort was stratified based on baseline eligibility for diabetes remission, defined as a diabetes duration ≤6 years and no insulin therapy. Cox proportional hazards models evaluated the effect of ILI, as compared with DSE, on the incidence of a composite CVD outcome, CKD, and all‐cause mortality.

The composite cardiovascular outcome was defined as per the primary outcome of the Look AHEAD trial, that is, a composite of death from cardiovascular causes, non‐fatal myocardial infarction, non‐fatal stroke, or hospitalisation for angina. CKD was defined as high‐risk or very high‐risk CKD, as per the Kidney Disease Improving Global Outcomes (KDIGO) criteria, that is, eGFR <45 mL/min irrespective of urine albumin to creatinine ratio (UACR); or eGFR <60 mL/min plus UACR ≥30 mg/g; or UACR >300 mg/g irrespective of eGFR.^13^

The models for CVD and mortality were adjusted for the presence of CVD at baseline. In the whole Look AHEAD population, there were two participants with high‐risk or very high‐risk CKD at baseline; they were excluded from the CKD models. Linear models with generalised least squares and structured covariance matrices were used to assess the effect of ILI on the trajectories of weight (kg), HbA1c (%), SBP (mmHg), LDL (mmol/L), and eGFR (mL/min). Logistic and Poisson regression were used to evaluate the effect of ILI on the use of medications and the number of medications, respectively, in the glucose‐lowering, lipid‐lowering, and blood pressure‐lowering classes. The effect of ILI on the risk of being on any medications of the given class was reported using odds ratios. The effect of ILI on the number of medications was reported using rate ratios (ratio of the average number of medications with ILI vs. with DSE alone, by the end of the follow‐up), obtained using Poisson regression.

R (version 4.4.0) was used for data management, statistical analyses, and figure production. A two‐sided *p*‐value <0.05 was considered statistically significant.

The Look AHEAD study was funded by the National Institutes of Health through cooperative agreements with the National Institute of Diabetes and Digestive and Kidney Diseases. This study was approved by the University of Melbourne Human Ethics Committee, Melbourne, Victoria, Australia (reference number 2024‐29189‐51684‐2).

## 3 RESULTS

### 3.1 Characteristics of participants

**FIGURE 1:**
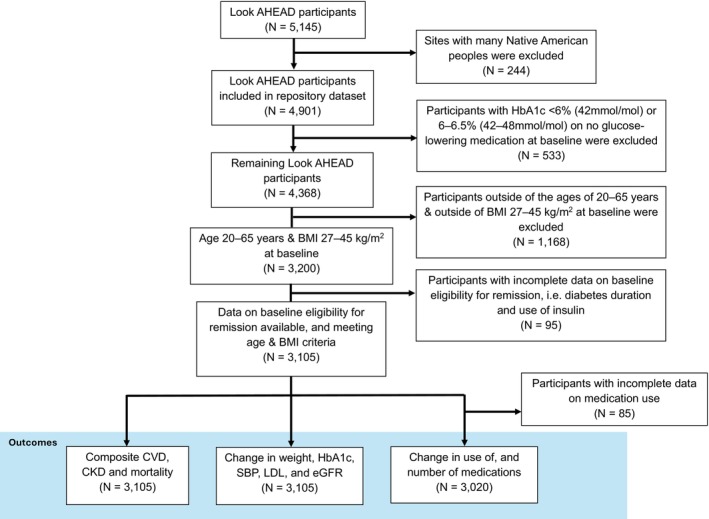
Flowchart depicting participant numbers for the restricted population analyses in this study. For each of the three main sets of outcomes, there were fewer than 3200 participants included, since not all participants had available data on eligibility for remission and/or data on the given outcome(s) at the start and end of follow‐up. CKD, chronic kidney disease; eGFR, estimated glomerular filtration rate; LDL, low‐density lipoprotein cholesterol; SBP, systolic blood pressure.

There were 3105 Look AHEAD participants eligible for inclusion when analysing the effect of ILI on composite CVD, CKD, and mortality, by eligibility for diabetes remission at baseline. A flowchart depicting participant selection is shown in Figure 1.

Among these 3105 participants, the median age (IQR) was 58.0 years (55.0–61.5 years), and 59.6% were women. Most participants were white (63.0%), while 17.0% were African American and 16.5% were Hispanic. The median duration of diabetes was 5 years (IQR 2–10 years). For the main trial period, the median duration of follow‐up was 9.0 years (IQR 8.1–10.0 years). Baseline characteristics of the study populations are shown in Table 1—within each of these populations, there were no significant differences between participants in the ILI versus DSE group.

**TABLE 1:** Baseline characteristics by study population.

	Unrestricted population (*N* = 4368)	Population restricted to DiRECT age and BMI criteria (*N* = 3200)		
		Restricted population without stratification	Stratified by eligibility for diabetes remission (*N* = 3105)	
			Eligible	Ineligible
Median age (years, IQR)				
ILI	59.1 (55.3–63.9)	57.9 (54.8–61.5)	57.2 (54.4–61.1)	58.7 (55.1–62.0)
DSE	59.4 (55.5–64.5)	58.0 (55.1–61.5)	57.7 (54.7–61.1)	58.7 (55.4–62.1)
Sex (women (%))				
ILI	1272/2172 (59)	949/1586 (60)	509/819 (62)	415/725 (57)
DSE	1291/2196 (59)	964/1614 (60)	523/856 (61)	403/705 (57)
Race (white (%))				
ILI	1409/2172 (65)	991/1586 (62)	533/819 (65)	435/725 (60)
DSE	1439/2196 (66)	1021/1614 (63)	544/856 (64)	445/705 (63)
Median BMI (kg/m^2^, IQR)				
ILI	34.9 (31.5–39.6)	34.8 (31.8–38.6)	34.8 (31.5–38.5)	34.9 (32.1–38.9)
DSE	35.3 (31.8–39.4)	35.2 (31.9–38.6)	35.5 (32.0–38.8)	34.8 (31.8–38.4)
Smoking status^a^ (ever smoker (%))				
ILI	1104/2168 (51)	796/1584 (50)	403/819 (49)	370/725 (51)
DSE	1086/2190 (50)	780/1612 (48)	405/856 (47)	350/705 (50)
Median diabetes duration (years, IQR)				
ILI	5 (2–10)	5 (2–10)	3 (1–5)	10 (7–14)
DSE	5 (3–10)	5 (2–10)	3 (1–5)	10 (8–14)
Median HbA1c (%, IQR)				
ILI	7.2 (6.6–8.0)	7.2 (6.7–8.0)	7.0 (6.5–7.6)	7.5 (6.9–8.4)
DSE	7.2 (6.7–8.0)	7.2 (6.7–8.1)	7.1 (6.6–7.8)	7.6 (6.9–8.5)
Median SBP (mmHg, IQR)				
ILI	128 (117–140)	126 (116–139)	127 (116–138)	126 (116–141)
DSE	130 (118–142)	128 (116–140)	128 (116–140)	128 (117–140)
Median LDL (mmol/L, IQR)				
ILI	2.8 (2.3–3.4)	2.8 (2.3–3.4)	2.9 (2.4–3.6)	2.8 (2.3–3.3)
DSE	2.9 (2.3–3.4)	2.9 (2.4–3.5)	2.9 (2.4–3.5)	2.8 (2.3–3.4)
Median eGFR (mL/min, IQR)				
ILI	93.0 (80.1–101.0)	95.1 (82.0–102.0)	95.1 (82.8–102.0)	94.5 (81.3–101.0)
DSE	92.4 (78.6–100.0)	94.8 (81.4–101.0)	95.5 (82.4–102.0)	93.8 (80.0–101.0)

Abbreviations: BMI, body mass index; DSE, diabetes support and education; eGFR, estimated glomerular filtration rate; ILI, intensive lifestyle intervention; IQR = interquartile range; LDL, low‐density lipoprotein cholesterol; SBP, systolic blood pressure.

^a^ In the unrestricted population, there were missing smoking status data for 4 participants in the ILI group and 6 participants in the DSE group.

### 3.2 Effect of ILI on composite CVD, CKD, and mortality, by remission eligibility status

**FIGURE 2:**
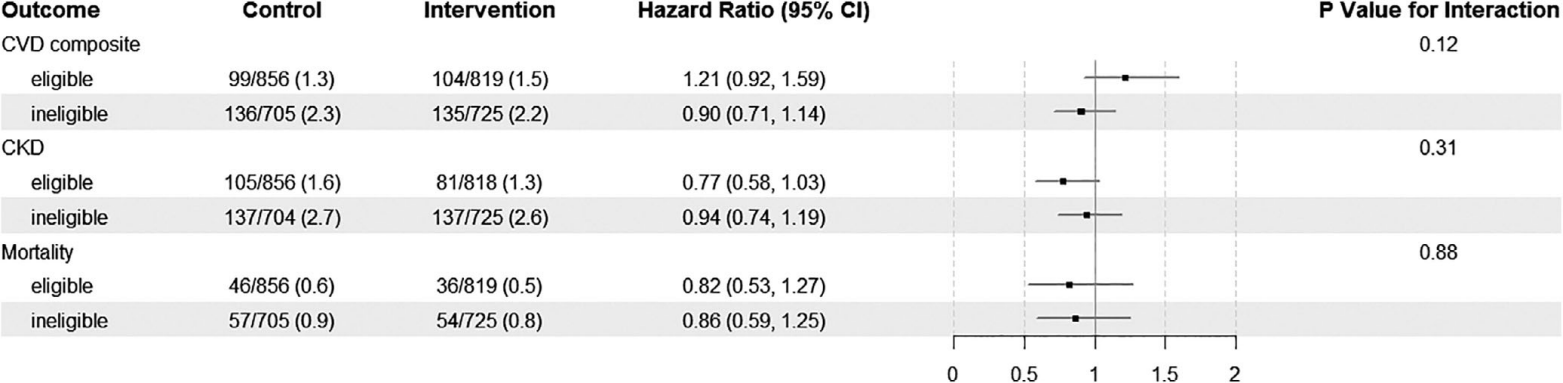
In the restricted population, the effect of the intensive lifestyle intervention, as compared with diabetes support and education, on CVD, CKD, and mortality in people who were and were not eligible for diabetes remission programmes at baseline. CVD, cardiovascular disease; CKD, chronic kidney disease; DSE, diabetes support and education; ILI, intensive lifestyle intervention; py, person‐years.

There were no significant benefits of ILI on composite CVD, CKD, or mortality in the restricted population or unrestricted population, except for CKD in the unrestricted population—here, ILI reduced the risk of incident CKD, as compared with DSE (hazard ratio 0.83 (95% CI 0.72, 0.96)) (Figure S1). After stratification by baseline eligibility for diabetes remission, there were no significant differences between the sub‐groups with regard to the effect of ILI on these outcomes (*p*‐values for interaction >0.05) (Figures 2 and S2).

### 3.3 Effect of ILI on weight and HbA1c, by remission eligibility status

**FIGURE 3:**
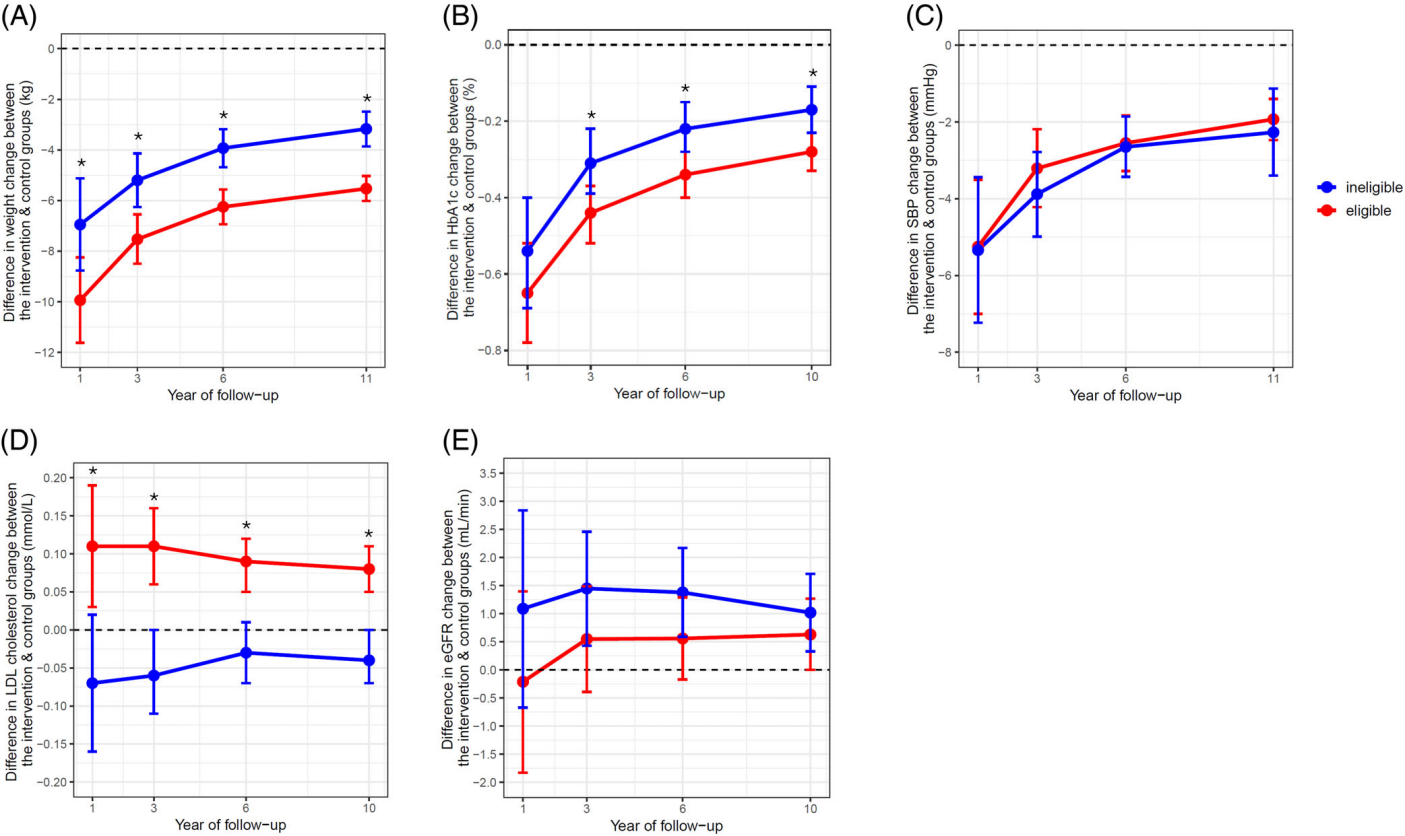
In the restricted population, the effect of the intensive lifestyle intervention, as compared with diabetes support and education, in people who were eligible (red) and were not eligible (blue) for diabetes remission programmes at baseline, with respect to change in (A) weight (kg), (B) HbA1c (%), (C) systolic blood pressure (SBP, mmHg), (D) LDL cholesterol (mmol/L), and (E) eGFR (mL/min), over the Look AHEAD trial period. Physical examination data were available up to 11 years, while biochemical data were available up to 10 years. Negative values indicate that the magnitude of the decrease in a given value was greater in the intervention group, as compared with the control group. Error bars represent 95% confidence intervals. * Statistically significant difference between the groups.

Overall, as compared with DSE, ILI led to improvements in weight, HbA1c, and SBP, and slowed the decline in eGFR. There was a minimal effect of ILI on LDL (Figure S3). In the restricted population, compared with people ineligible for diabetes remission at baseline, eligible people experienced on average a 2.4 kg greater weight loss (−5.53 kg (95% CI −6.02 kg, −5.03 kg) vs. −3.17 kg (95% CI −3.86 kg, −2.49 kg)), and a 0.1% greater HbA1c reduction (−0.28% (95% CI −0.33%, −0.23%) vs. −0.17% (95% CI −0.23%, −0.11%)) from ILI compared with DSE at 10–11 years (Figure 3). The HbA1c benefits of ILI diminished at a similar rate in both the eligible and non‐eligible populations. In the unrestricted population, there were no significant differences between eligible and ineligible people in terms of the impact of ILI on weight loss or HbA1c (Figure S5).

### 3.4 Effect of ILI on weight and HbA1c, by remission eligibility status—age‐restricted and BMI‐restricted populations

Given there was a significant difference in the impact of ILI on weight and HbA1c between the restricted and unrestricted populations in terms of eligibility groups, two additional analyses were performed. Weight and HbA1c were analysed in a population restricted to the DiRECT age criterion only (i.e., 20–65 years old), and a population restricted to the DiRECT BMI criterion only (i.e., 27–45 kg/m^2^). In the population restricted to the DiRECT BMI criterion only, ILI was more effective than DSE in reducing weight in eligible people, as compared with ineligible people (Figure S4). In the population restricted to the DiRECT age criterion only, as per the unrestricted population, there was no difference between eligible and ineligible people in terms of the impact of ILI on weight loss.

### 3.5 Effect of ILI on LDL, SBP, and eGFR, by remission eligibility status

In the restricted population, compared with people ineligible for diabetes remission at baseline, eligible people ended up with, on average, a 0.12 mmol/L greater LDL level (0.08 mmol/L (95% CI 0.05 mmol/L, 0.11 mmol/L) vs. −0.04 mmol/L (95% CI −0.07 mmol/L, 0.00 mmol/L)) from ILI compared with DSE at 10 years (Figure 3). A similar trend was observed in the unrestricted population (Figure S5).

In the restricted population, baseline eligibility for diabetes remission did not impact the effect of ILI on SBP or eGFR (Figure 3). In the unrestricted population, there was a lesser eGFR decline with ILI observed among ineligible people at 3 and 6 years of follow‐up (Figure S5).

### 3.6 Effect of ILI on medication use, by remission eligibility status

As compared with DSE, ILI reduced the use of, and number of, glucose‐lowering, blood pressure‐lowering, and cholesterol‐lowering medications (Figure S6). However, stratifying by baseline eligibility for diabetes remission did not impact the effect of ILI on the use of, or number of, glucose‐lowering, cholesterol‐lowering, or blood pressure‐lowering medications, at the start versus the end of the trial (Figures S7A,B).

## 4 DISCUSSION

Diabetes remission programmes are cost‐effective, but exclusively allocate ILI resources to people with type 2 diabetes of a shorter duration. This is despite these resources potentially benefiting people with a longer duration of type 2 diabetes to a similar degree. The current study suggests that this may not be justified. People with a longer duration of diabetes are able to achieve similar improvements in cardiometabolic parameters, as compared with people with a shorter duration of diabetes, and they are at a higher cardiovascular risk. Further research is required to determine the best way to distribute resources dedicated to cost‐effective ILI programmes, but the current method is likely not it.

ILI reduced the incidence of CKD in the unrestricted population, as described in prior studies,^14^ but it did not impact composite CVD or mortality. There were also no significant differences between the two eligibility groups with respect to the benefit of ILI for composite CVD, CKD, or mortality.

Regarding weight loss and HbA1c reduction, ILI confers a small additional benefit for people eligible for diabetes remission compared with ineligible people. The mean 2.4 kg difference in weight loss and 0.1% difference in HbA1c between eligible and ineligible groups in this study are not clinically significant. For example, guidelines consider 5%–10% weight loss to be significant.^15^ Similar to the current study, a 2012 randomised controlled trial of people post bariatric surgery found that diabetes duration was not associated with the degree of subsequent glycaemic improvement.^16^ Indeed, people with a longer duration of diabetes can improve their glycaemia and may achieve diabetes remission, although the underlying pathology does not revert to normal.^17^

Interestingly, this difference in the impact of ILI on weight and HbA1c between eligible and ineligible people disappeared when considering the unrestricted population; the differences persisted in the BMI‐restricted population, but not the age‐restricted population. This suggests that, outside of BMI 27–45 kg/m^2^, diabetes duration and insulin dependence are not significant predictors of weight response to ILI. It is noted that the Look AHEAD cohort was predominantly Caucasian. Healthy BMI ranges differ by ethnicity,^18^ so the findings may have differed in a more diverse population.

It is noted that there was a statistically significant difference in LDL at the end of the trial period between the eligibility groups, when comparing ILI with DSE. In people eligible for remission, the LDL outcome favoured DSE over ILI. However, while this difference was statistically significant, it was very small; compared with DSE, the ILI groups recorded an average of a 0.10–0.12 mmol/L difference in LDL between the eligibility groups at 10 years. Given this was a post hoc analysis, unmeasured confounding factors may account for this difference, or it may be due to chance.

This study suggests that the benefits of the intensive lifestyle interventions that underpin diabetes remission programmes are not restricted to those with a short duration of diabetes. Whilst remission of diabetes seems an attractive goal, improvement in metabolic parameters and reduction in medication use, especially insulin, are also potentially valuable outcomes of ILI programmes that are relevant at later stages of type 2 diabetes. Thus, an important implication of these findings is the need to identify factors that predict response to ILI. These may be strictly biological or may relate to preparedness to adopt lifestyle change.

This is the first study to investigate the impact of eligibility for diabetes remission on cardiometabolic outcomes achieved through ILI, outside of remission. Bancks et al. found that there was no significant difference between people with longer and shorter durations of diabetes with regard to the impact of ILI on cardiovascular disease and death.^19^ However, these analyses were performed for a different purpose to the current study and therefore, were not adjusted for age; the longer duration of diabetes group was 15 years older, so the effects of diabetes duration versus age cannot be distinguished. Further, other cardiometabolic outcomes, such as HbA1c, weight, LDL, and eGFR, were not assessed.

The current study has limitations. First, data on participants' history of bariatric surgery were not available in the repository dataset, so we were unable to adjust for any effects that bariatric surgery may have had on the examined outcomes. If participants who received bariatric surgery were not evenly distributed between the ILI and DSE groups, this could have confounded the results. In the original Look AHEAD trial, 4.9% of participants had a history of bariatric surgery.^20^ Second, this was a post‐hoc analysis—the randomisation did not account for the stratifications we used and there may still have been unmeasured confounding factors, such as socioeconomic position, ethnicity, and comorbidities. However, after stratification by eligibility, the groups were well‐balanced with regard to measured characteristics. Furthermore, we adjusted for baseline CVD for the composite CVD and mortality outcomes, and excluded participants with CKD at baseline when evaluating incident CKD. Third, the Look AHEAD trial intervention did not benefit CVD or mortality, potentially rendering it difficult to identify sub‐group differences in responses for these outcomes. This intervention was also not specifically a diabetes remission intervention; it was originally designed to assess the effect of weight loss on CVD outcomes. An intervention specifically targeting diabetes remission may have produced different results, although the Look AHEAD ILI was still more likely to induce remission than was DSE.^3^ Finally, the binary definition of eligibility for diabetes remission used did not incorporate measures of β‐cell function or insulin resistance; incorporating such metrics may have led to different results. However, these metrics were not available in the Look AHEAD dataset, and diabetes duration and insulin use were more practical.

This study also has key strengths. First, the large population and the broad range of baseline characteristics allowed a robust analysis of the benefits of ILI across different stages of diabetes. Second, the long follow‐up in Look AHEAD provided an opportunity to examine the impact of ILI over a prolonged period of time; potential alterations to the natural history of diabetes resulting from early initiation of ILI, rather than just short‐term changes in HbA1c, could be observed.

In summary, while there was a slightly greater weight loss and HbA1c reduction for people eligible for diabetes remission, the differences were small. Other outcomes did not differ among those who were and were not eligible for remission. These findings suggest that restricting ILI programmes to people eligible for remission may not be justified. It also demonstrates that people with a longer duration of diabetes can improve cardiometabolic parameters, just like people with a shorter duration of diabetes.

If ILI cannot be offered to all people with type 2 diabetes, then further research is required to identify more effective selection criteria for these cost‐effective ILI programmes. The current inclusion criteria do not effectively select the people with type 2 diabetes who will benefit the most from ILI; the current people being included attain a similar degree of cardiometabolic benefit as compared with the people who are missing out.

## CONFLICT OF INTEREST STATEMENT

JES has received consulting and/or lecturing honoraria from Abbott, Astra Zeneca, Boehringer Ingelheim, Eli Lilly, GlaxoSmithKline, Novo Nordisk, Roche, and Sanofi.

## PEER REVIEW

The peer review history for this article is available at https://www.webofscience.com/api/gateway/wos/peer-review/10.1111/dom.16650.

## Supporting information


**Data S1.** Figures.

## Data Availability

Data from the Look AHEAD trial are available for request at the NIDDK Central Repository (NIDDK‐CR) website, Resources for Research (R4R), https://repository.niddk.nih.gov/.
