# Supplementary material for: Impact of eligibility for diabetes remission on response to intensive lifestyle intervention in overweight and obese people with type 2 diabetes: The Look AHEAD trial
Source: Diabetes Obes Metab. 2025 Jul 24;27(10):5930–7. doi: 10.1111/dom.16650 (PMC12409236; doi:10.1111/dom.16650)
Supplement: Supplementary file 1 — Data S1. Figures. [file DOM-27-5930-s001.docx]

**Supplemental Material**

**
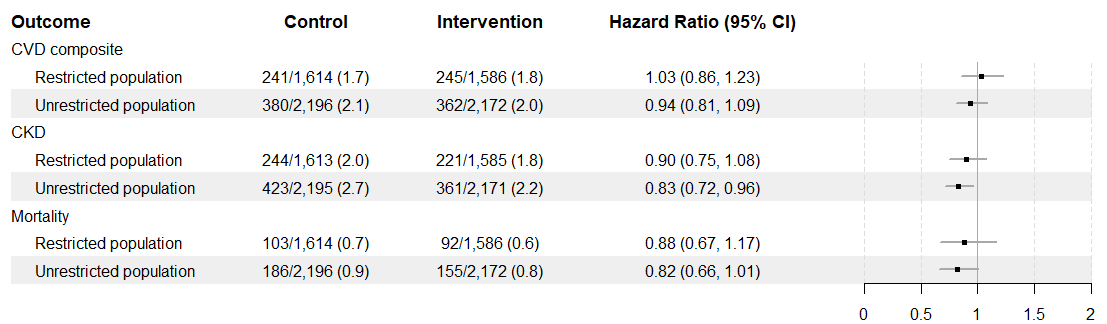
**

*no. of events (rate/100 py)*

favors ILI

favors DSE

**Figure S1.** In the restricted and unrestricted populations, the effect of the intensive lifestyle intervention, as compared with diabetes support and education, on CVD, CKD, and mortality. CKD = chronic kidney disease; ILI = intensive lifestyle intervention; DSE = diabetes support and education; py = person-years.

**
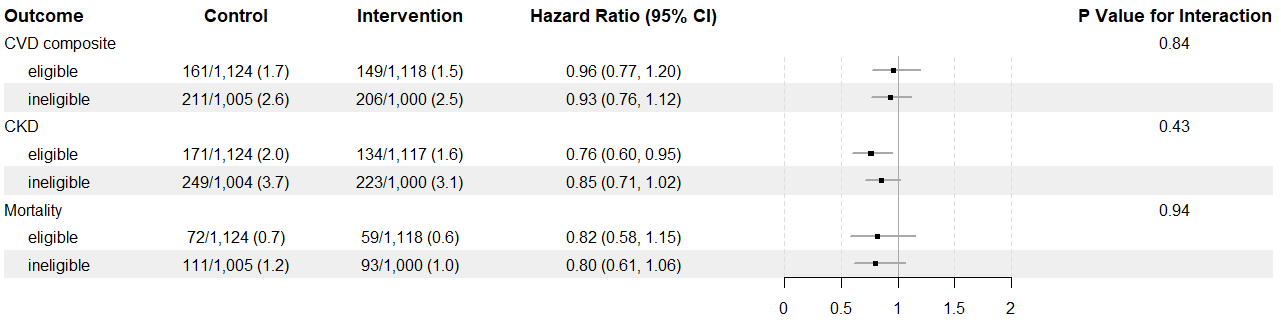
**

favors ILI

favors DSE

*no. of events (rate/100 py)*

**Figure S2.** In the unrestricted population, the effect of the intensive lifestyle intervention, as compared with diabetes support and education, on CVD, CKD, and mortality, in people who were and were not eligible for diabetes remission programs at baseline. CKD = chronic kidney disease;
ILI = intensive lifestyle intervention; DSE = diabetes support and education; py = person-years.

a) b) c)


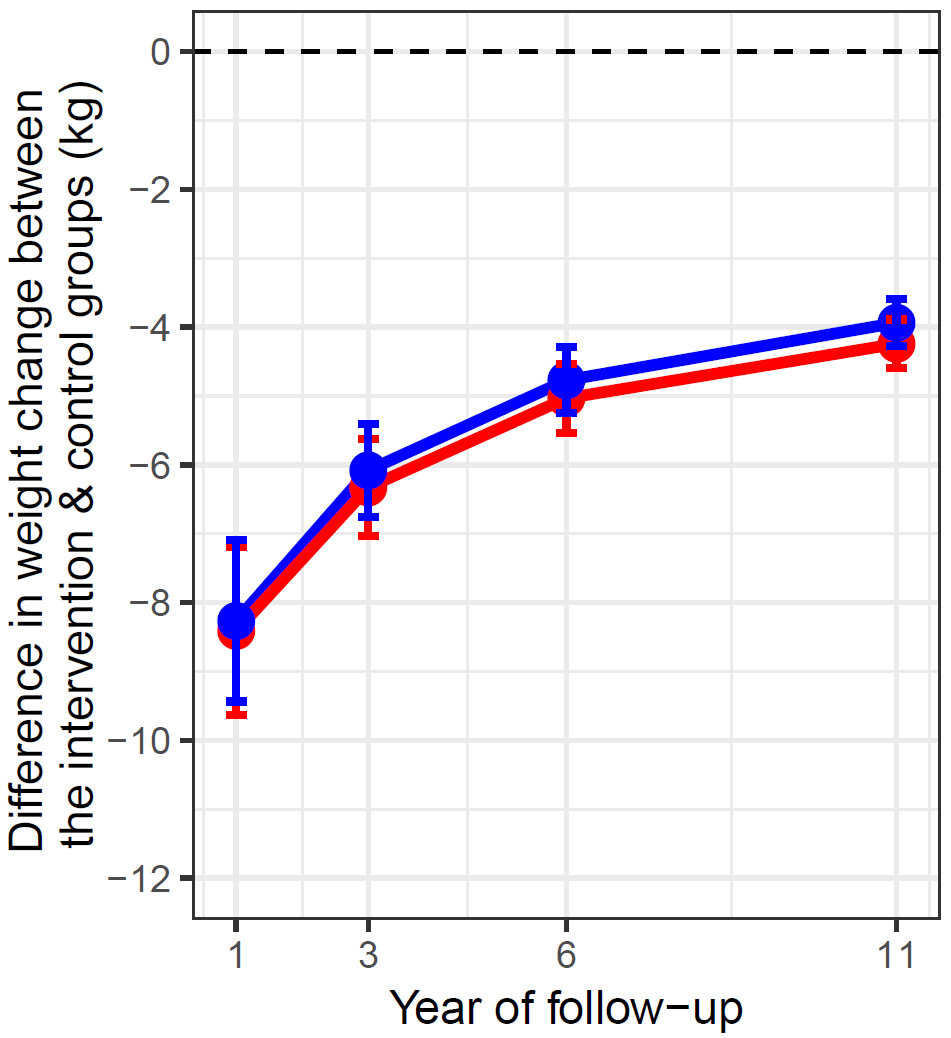

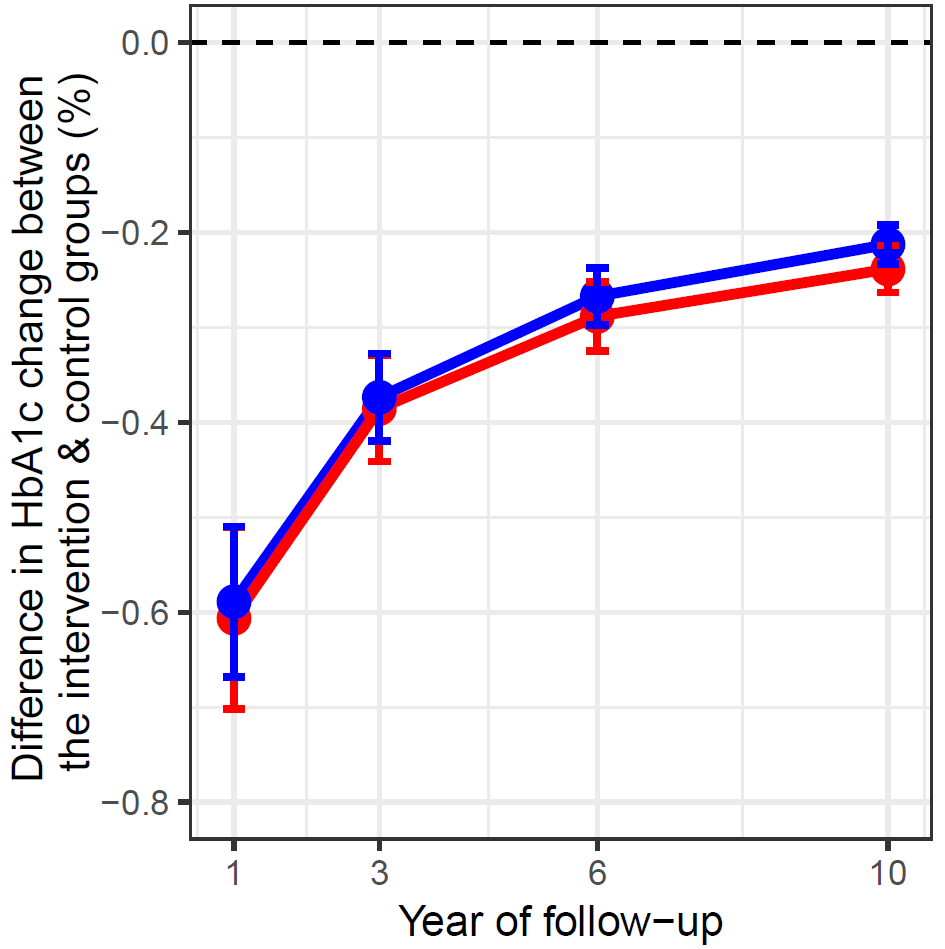

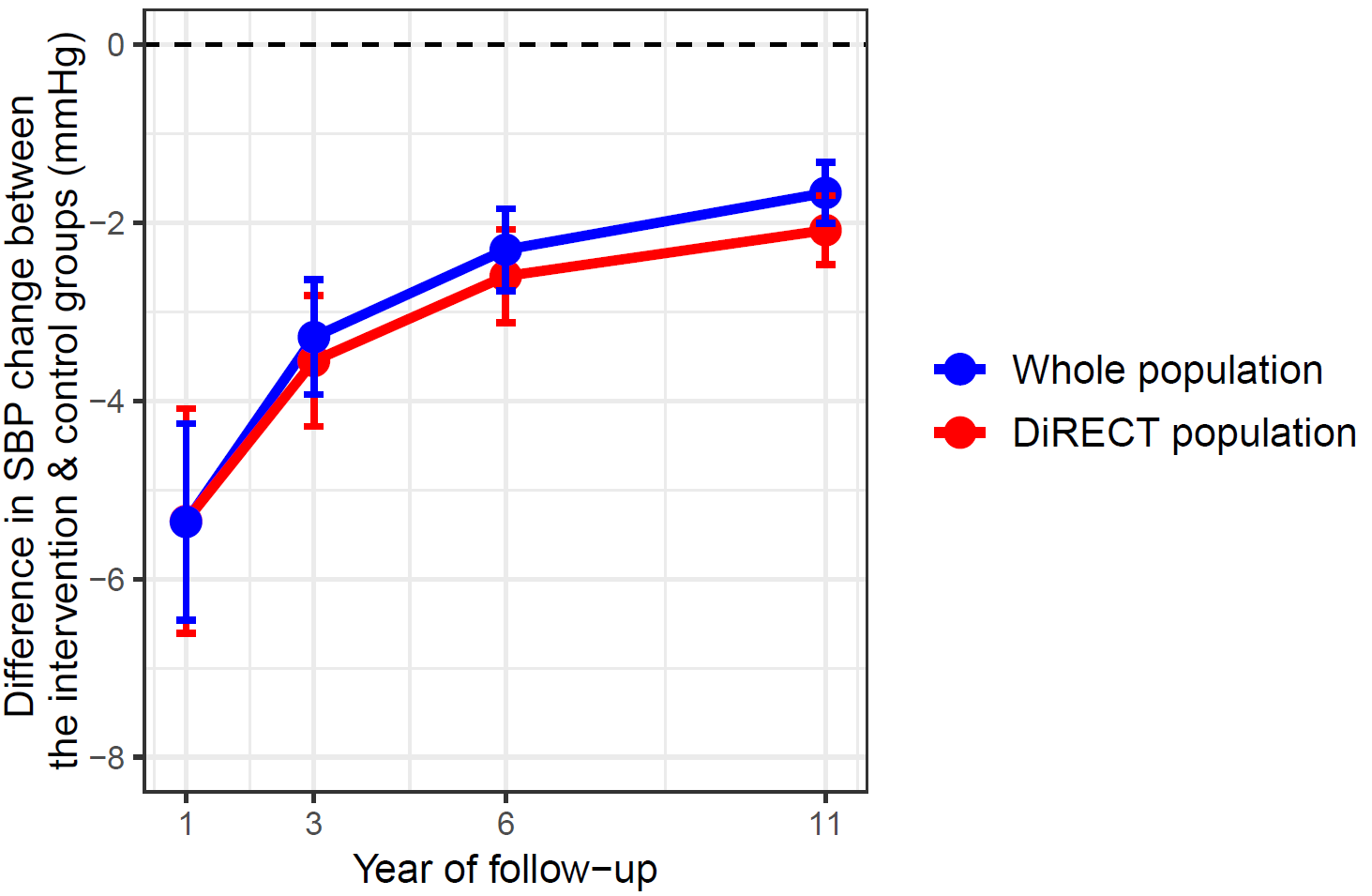


Unrestricted population
Restricted population

d) e)


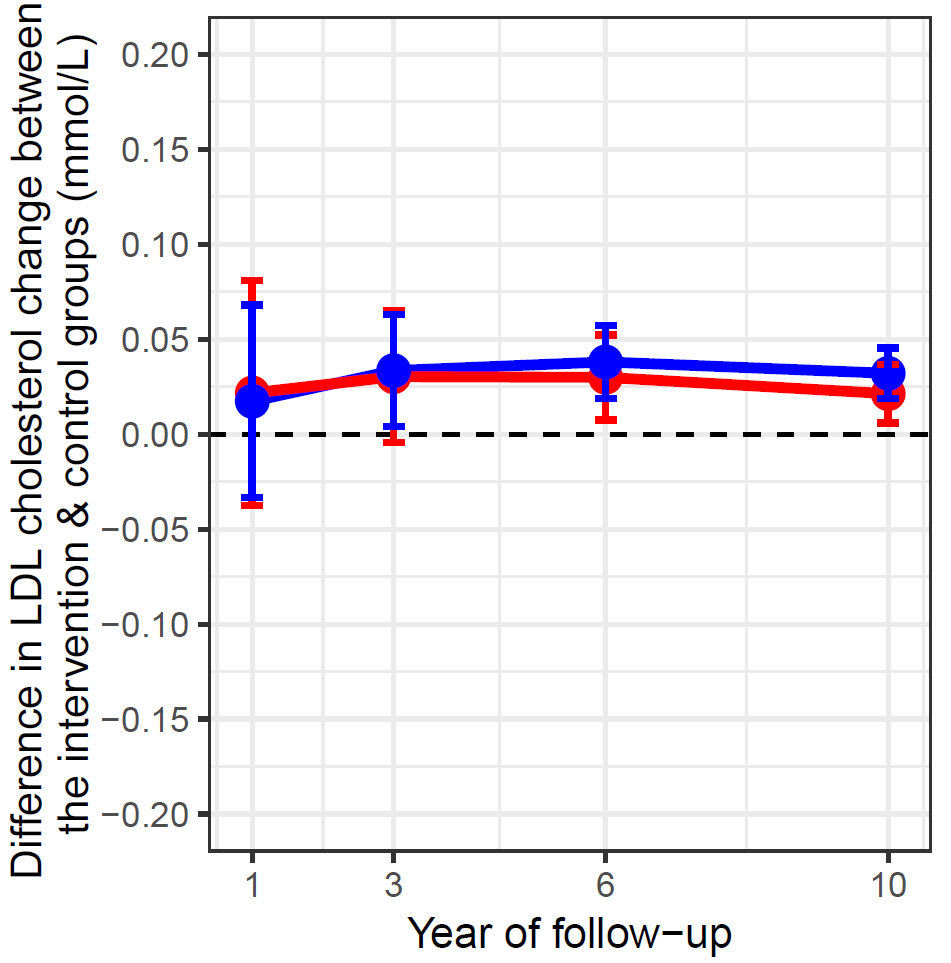

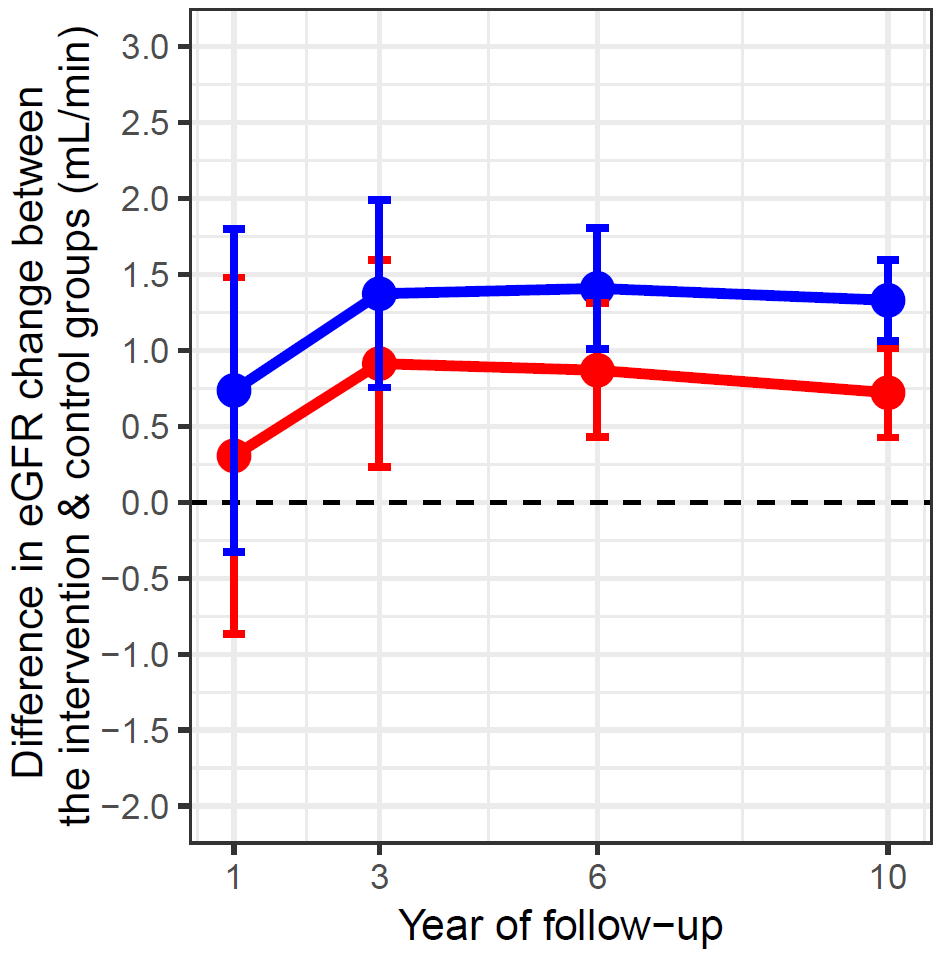


*

**Figure S3.** In the restricted (red) and unrestricted populations (blue), the effect of the intensive lifestyle intervention, as compared with diabetes support and education, with respect to change in a) weight (kg), b) HbA1c (%), c) systolic blood pressure (SBP, mmHg), d) LDL cholesterol (mmol/L), and e) eGFR (mL/min), over the Look AHEAD trial period. Physical examination data were available up to 11 years, while biochemical data were available up to 10 years. Negative values indicate that the magnitude of the decrease in a given value was greater in the intervention group, as compared with the control group. Error bars represent 95% confidence intervals. * Statistically significant difference between the groups.

a) b)


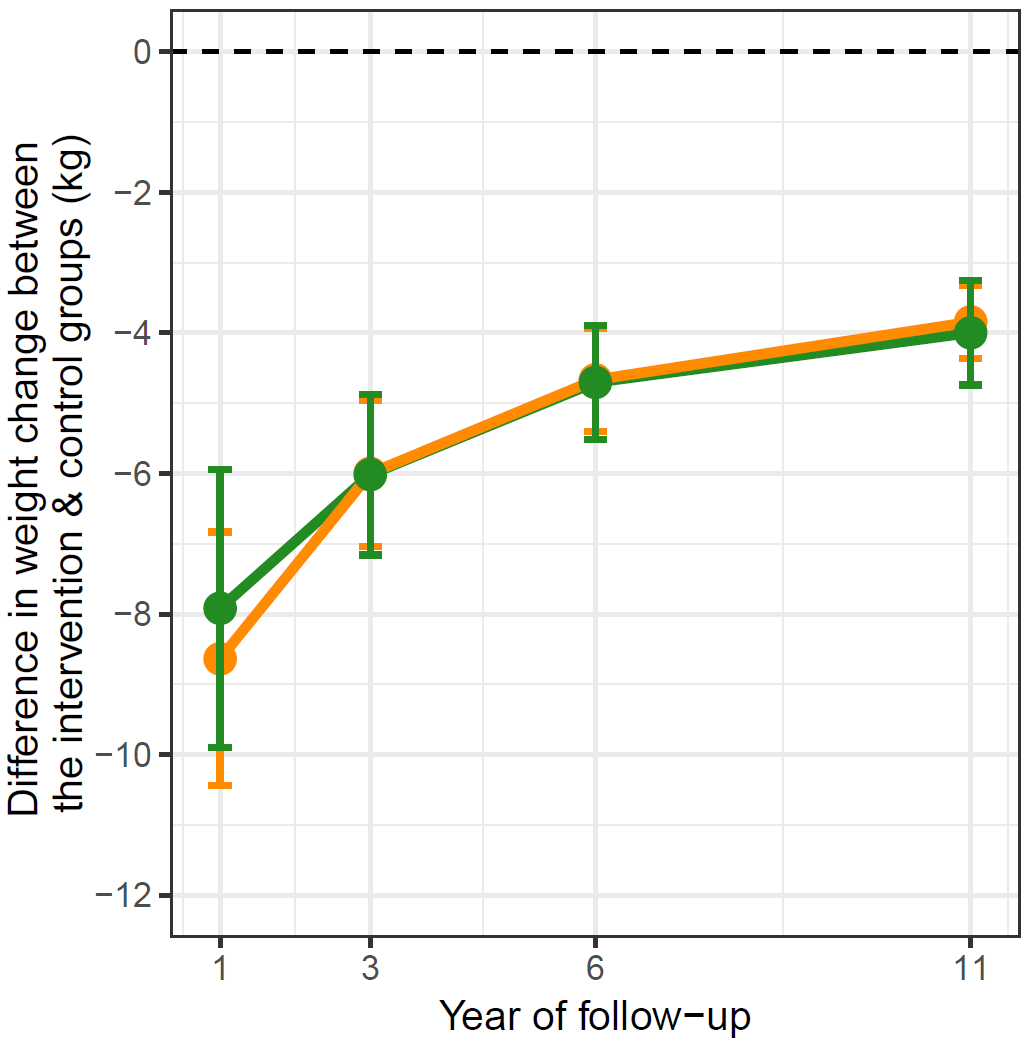

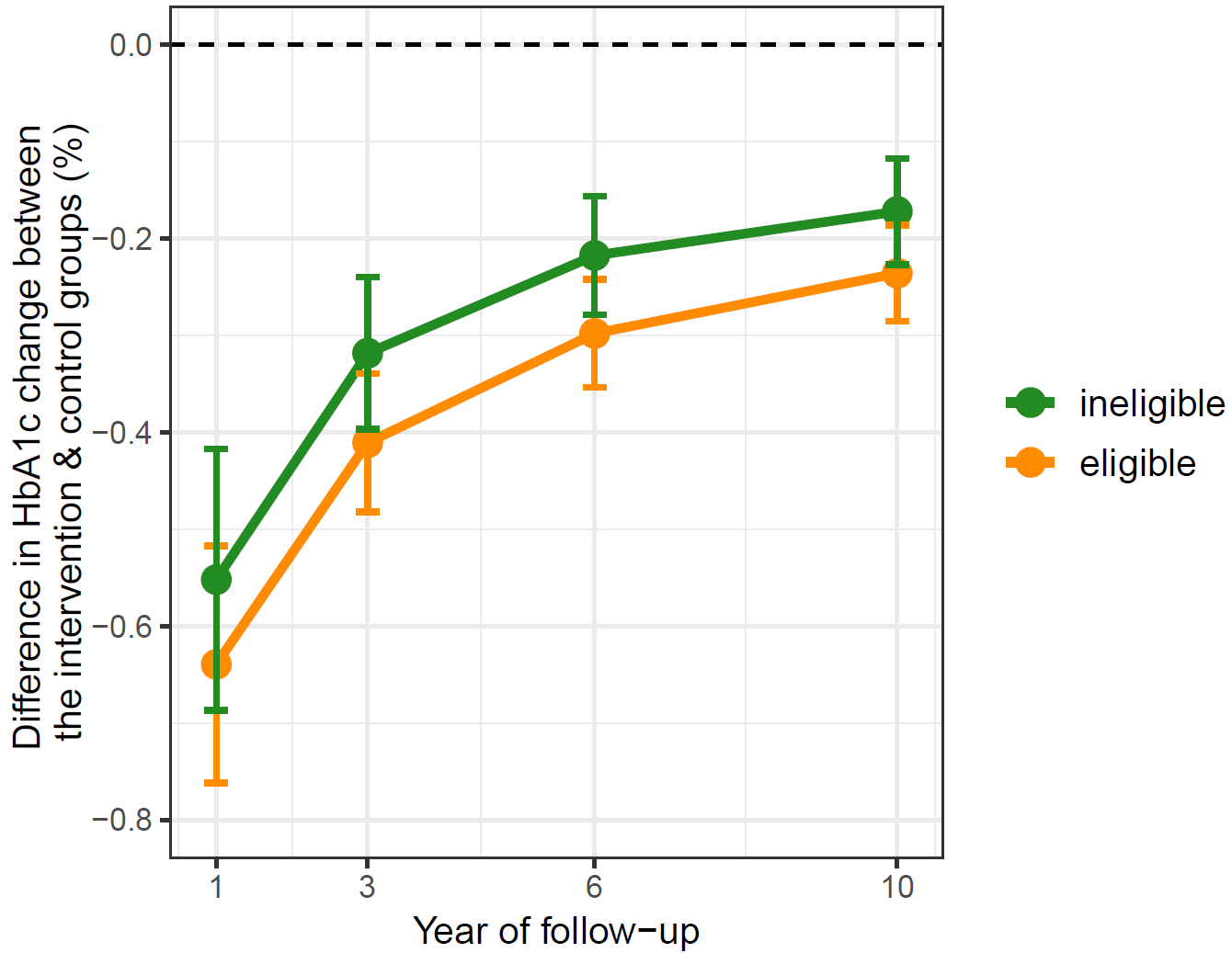


c) d)


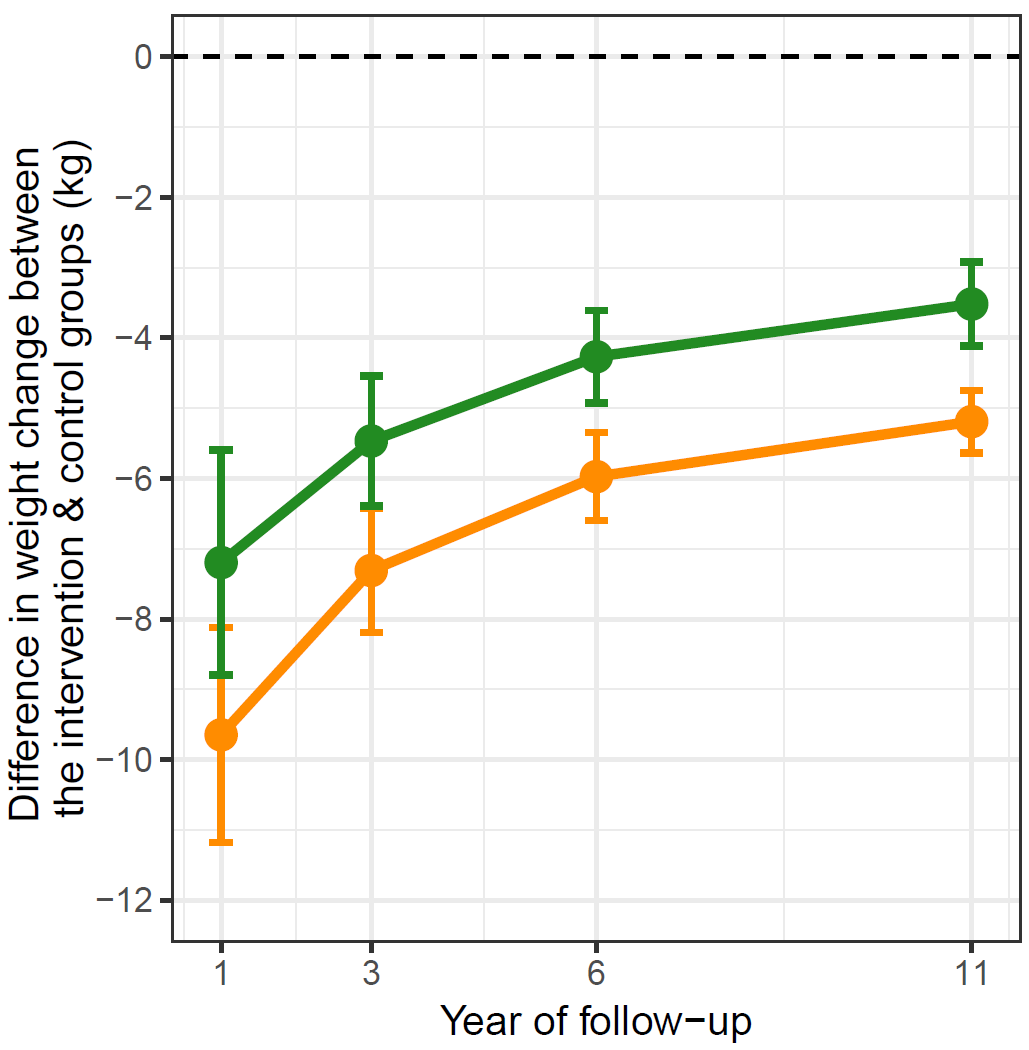

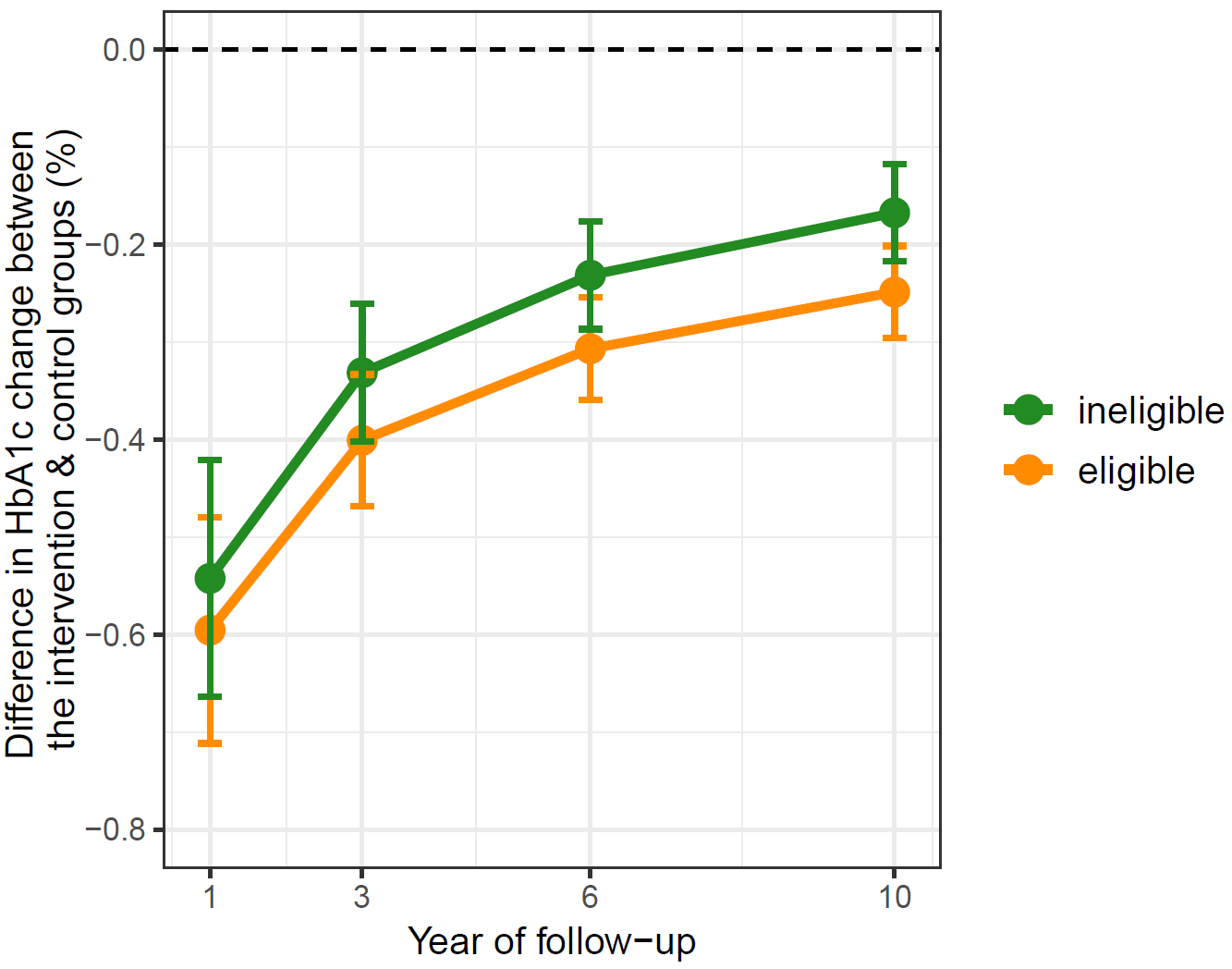


*

*

*

*

*

**Figure S4.** The effect of the intensive lifestyle intervention, as compared with diabetes support and education, in people who were eligible (orange) and were not eligible (green) for diabetes remission programs at baseline, with respect to change in a) and c) weight (kg), and b) and d) HbA1c (%), over the Look AHEAD trial period. The population represented in the top row (panels a and b) is restricted to the DiRECT age criterion, i.e. 20–65 years old. The population represented in the bottom row (panels c and d) is restricted to the DiRECT BMI criterion, i.e. 27‒45 kg/m^2^. Physical examination data were available up to 11 years, while biochemical data were available up to 10 years. Negative values indicate that the magnitude of the decrease in a given value was greater in the intervention group, as compared with the control group. Error bars represent 95% confidence intervals. * Statistically significant difference between the groups.

a) b) c)


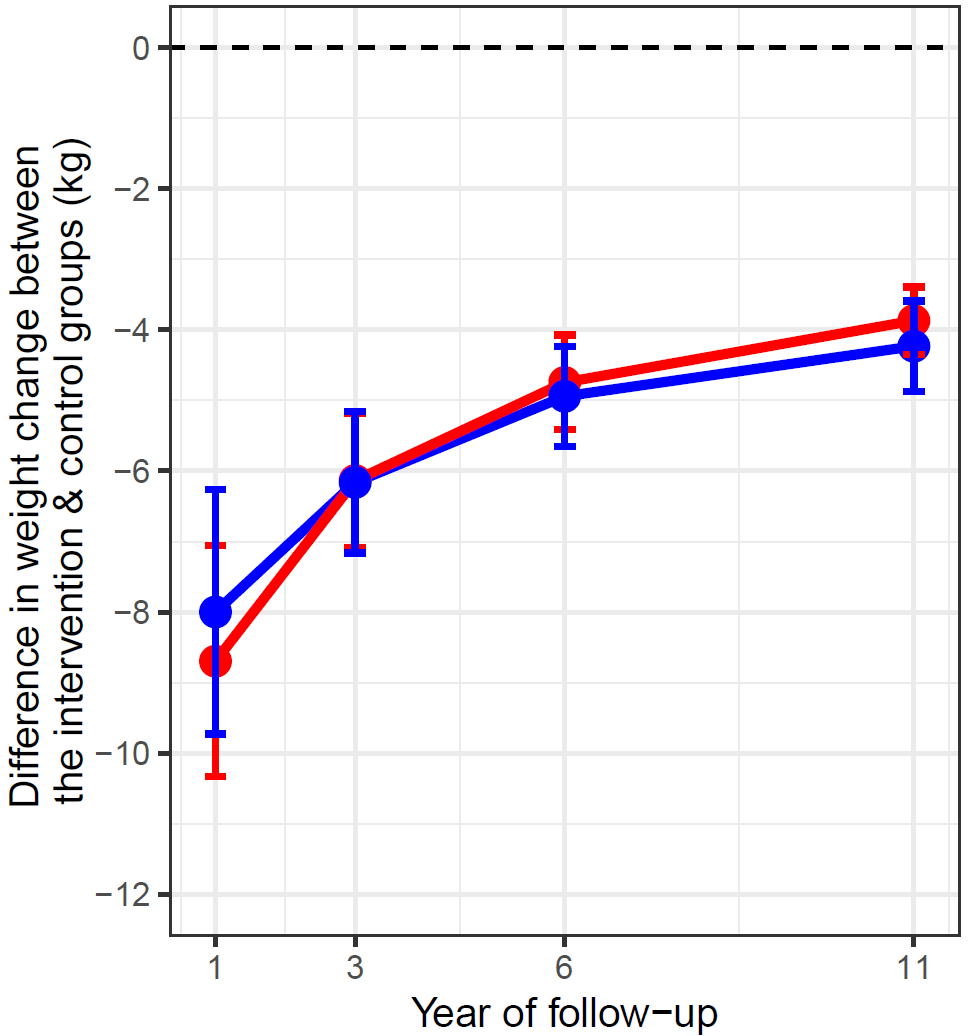

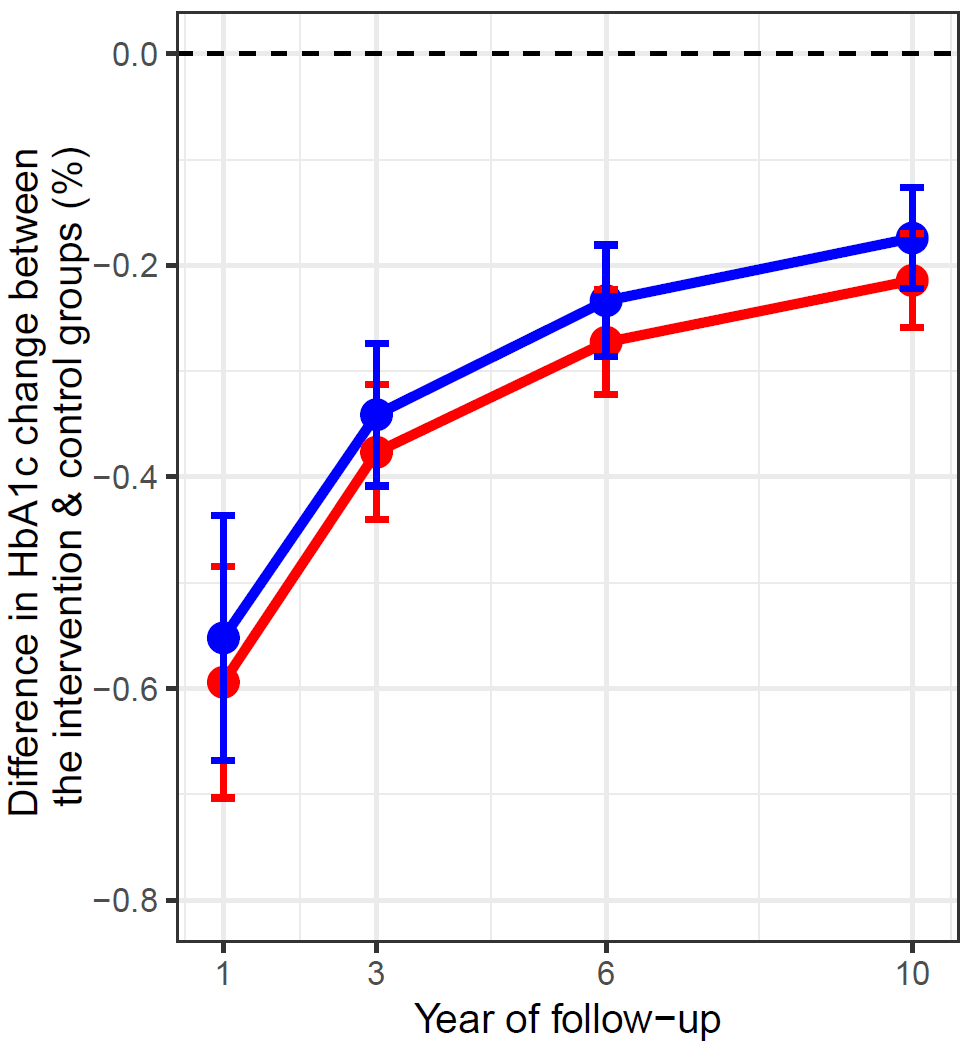

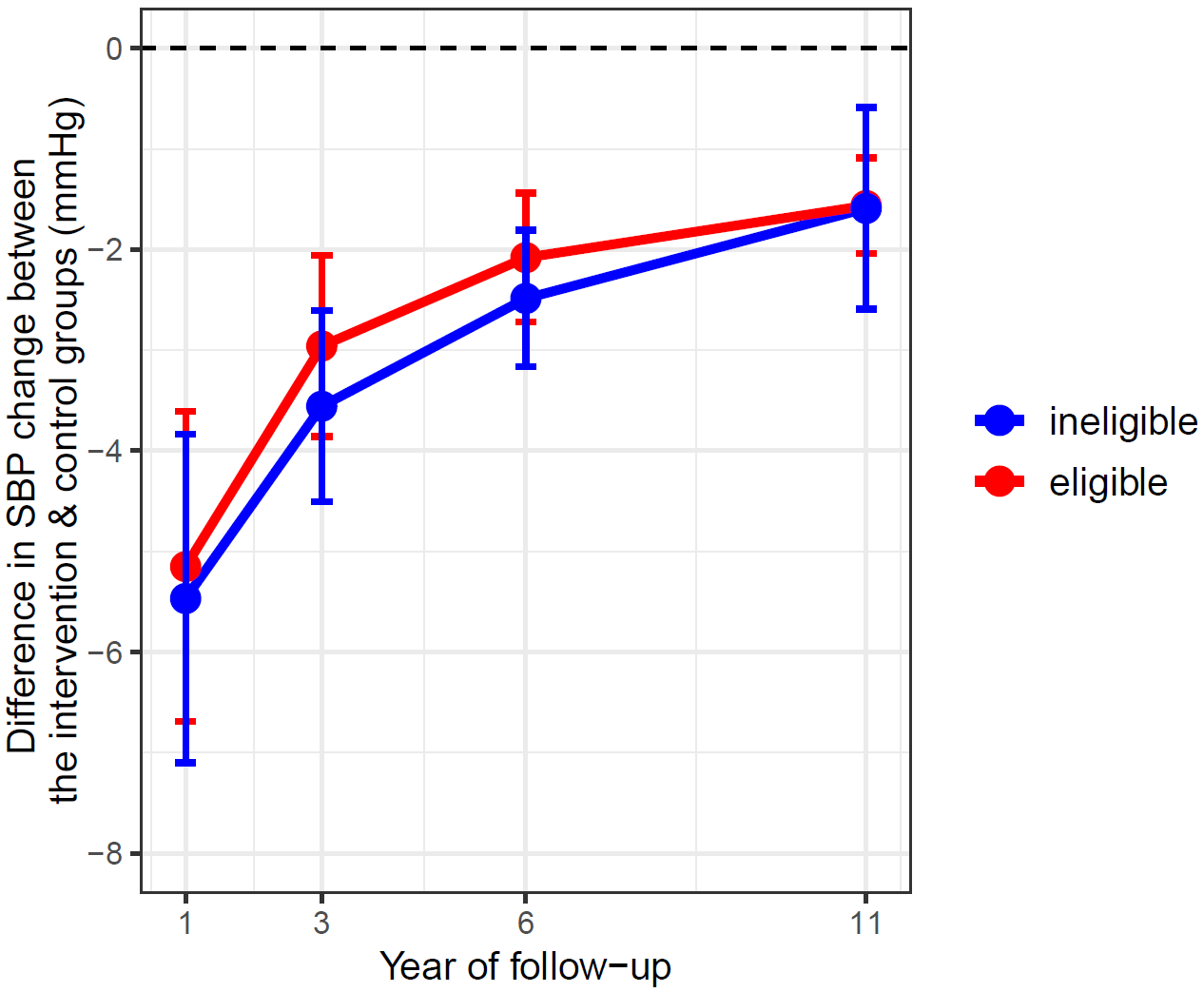


d) e)


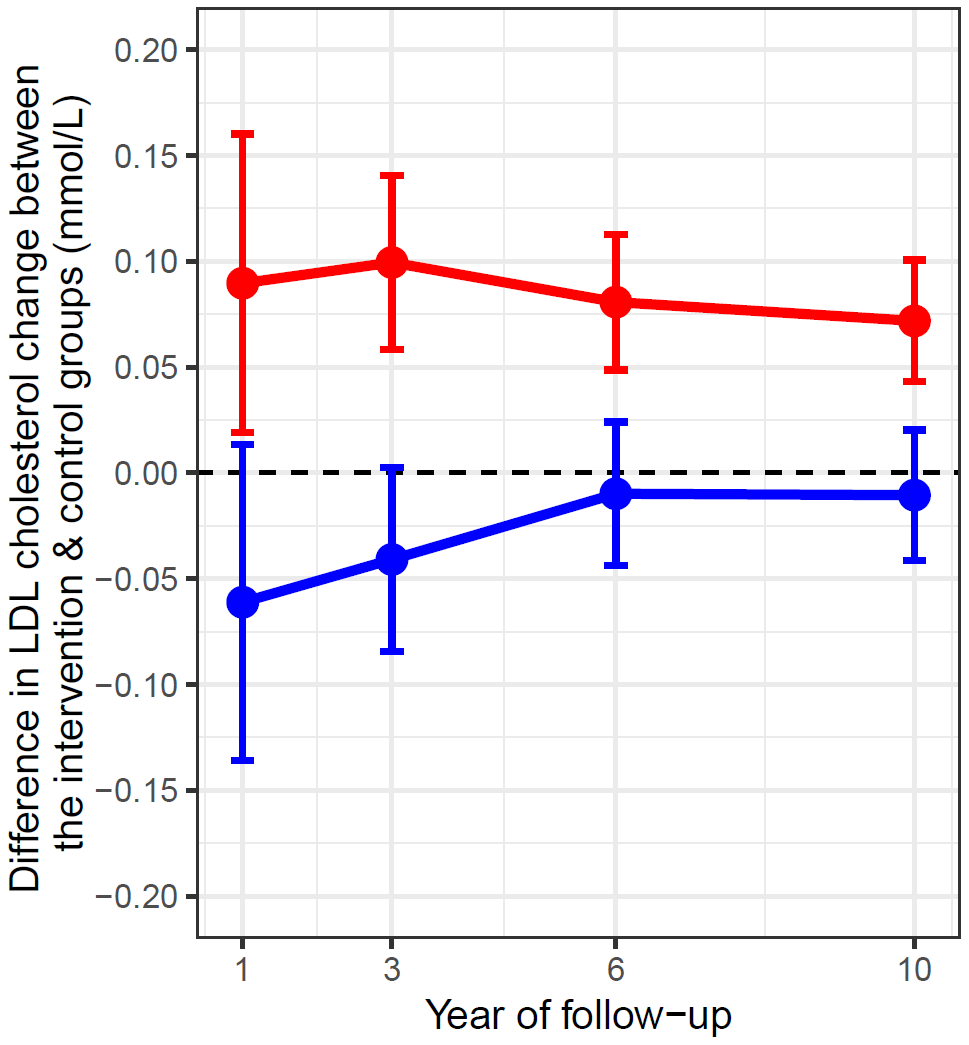

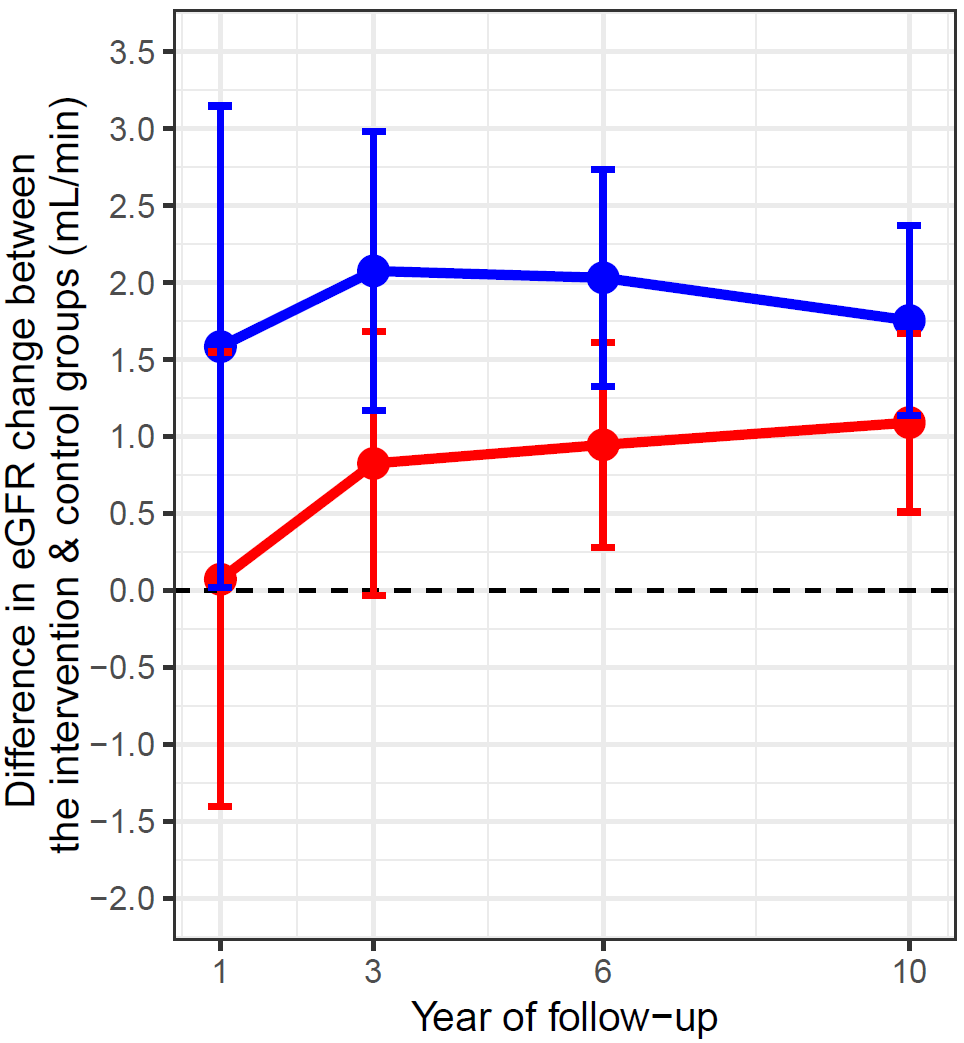


*

*

*

*

*

*

**Figure S5.** In the unrestricted population, the effect of the intensive lifestyle intervention, as compared with diabetes support and education, in people who were eligible (red) and were not eligible (blue) for diabetes remission programs at baseline, with respect to change in a) weight (kg), b) HbA1c (%), c) systolic blood pressure (SBP, mmHg), d) LDL cholesterol (mmol/L), and e) eGFR (mL/min), over the Look AHEAD trial period. Physical examination data were available up to 11 years, while biochemical data were available up to 10 years. Negative values indicate that the magnitude of the decrease in a given value was greater in the intervention group, as compared with the control group. Error bars represent 95% confidence intervals. * Statistically significant difference between the groups.

a)


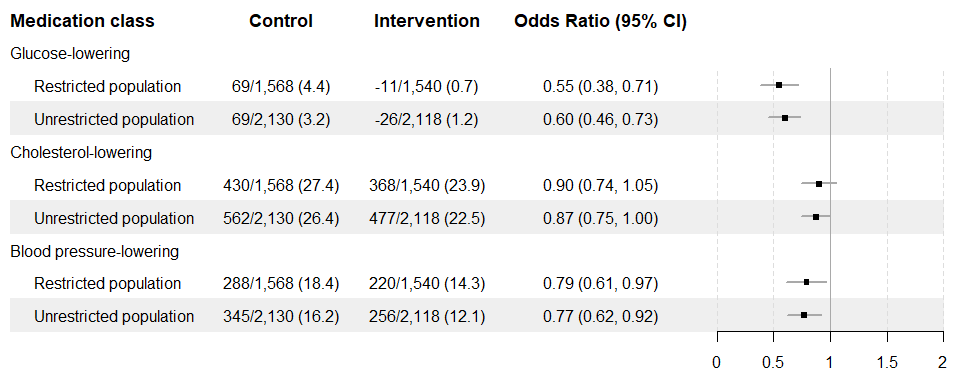


favors ILI

favors DSE

*net change/no. of participants (%)*

b)

**
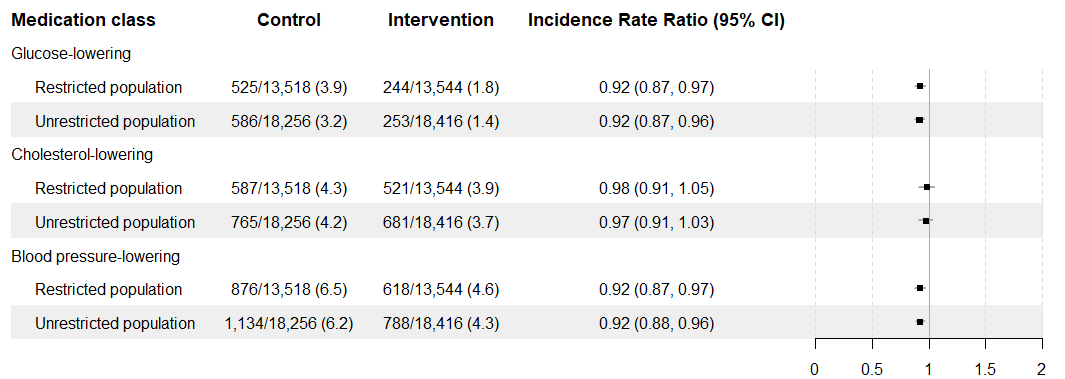
**

favors ILI

favors DSE

*net change/py (rate/100 py)*

**Figure S6.** In the restricted and unrestricted populations, the effect of the intensive lifestyle intervention, as compared with diabetes support and education, on a) the use of medications as a binary outcome (odds ratios represent the risk of being on any medications of the given class by the end of follow-up), and b) the number of medications within each of three classes, at the start versus the end of the Look AHEAD trial period (incidence rate ratios represent the risk of increasing the medication number by one by the end of follow-up). ‘Net change/py’ represents the net change in medications in the given population, over the number of person-years of follow-up in that population – negative numbers are observed given that participants could stop medications. ILI = intensive lifestyle intervention; DSE = diabetes support and education; py = person-years.

a)

**
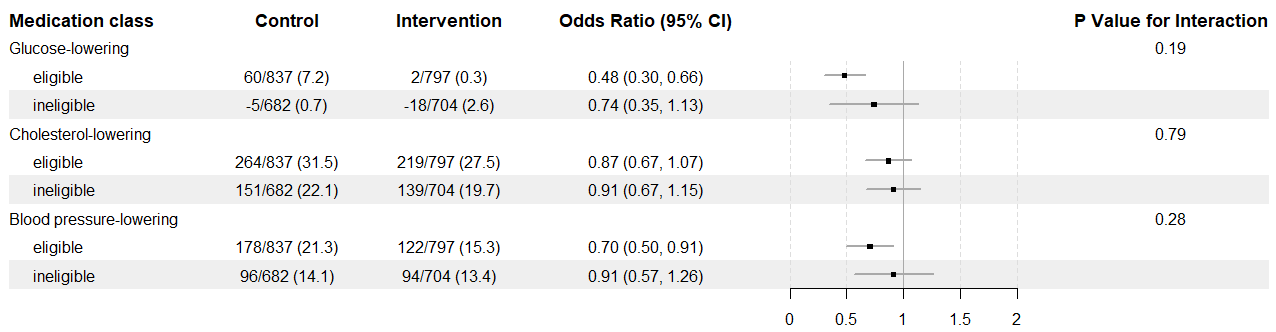
**

*net change/no. of participants (%)*

b)

favors ILI

favors DSE

**
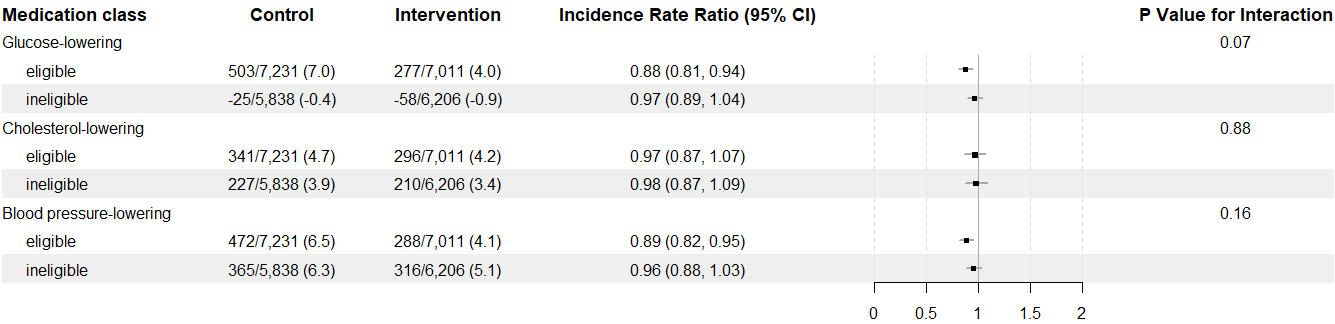
**

*net change/py (rate/100 py)*

favors ILI

favors DSE

**Figure S7A.** In the restricted population, the effect of the intensive lifestyle intervention, as compared with diabetes support and education, on a) the use of medications as a binary outcome (odds ratios represent the risk of being on any medications of the given class by the end of follow-up), and b) the number of medications within each of three classes, at the start versus the end of the Look AHEAD trial period (incidence rate ratios represent the risk of increasing the medication number by one by the end of follow-up), in people who were and were not eligible for diabetes remission programs at baseline. ‘Net change/py’ represents the net change in medications in the given population, over the number of person-years of follow-up in that population – negative numbers are observed given that participants could stop medications. ILI = intensive lifestyle intervention; DSE = diabetes support and education; py = person-years.

a)


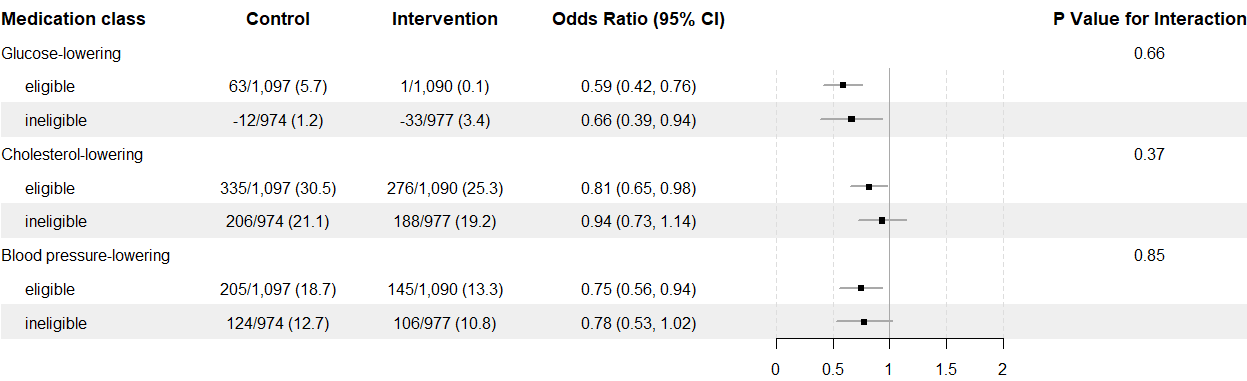


*net change/no. of participants (%)*

b)

favors ILI

favors DSE


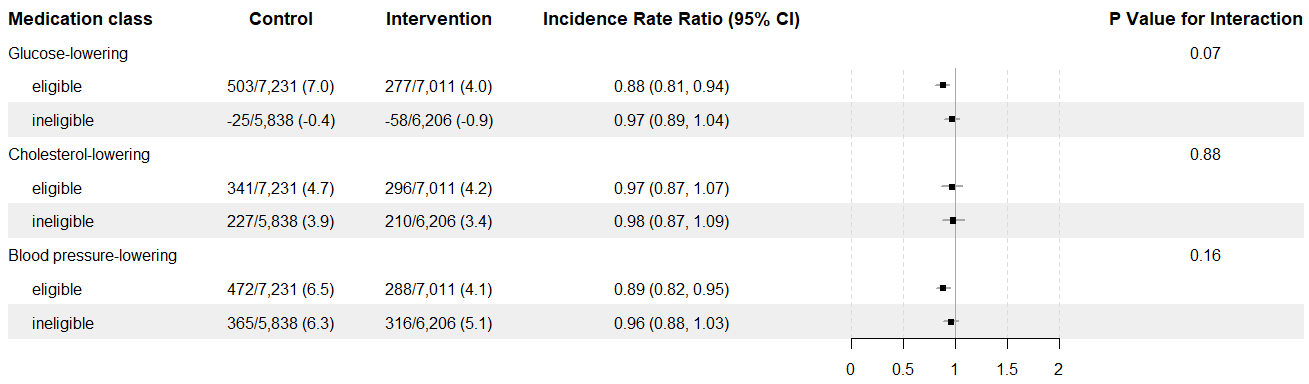


*net change/py (rate/100 py)*

favors ILI

favors DSE

**Figure S7B.** In the unrestricted population, the effect of the intensive lifestyle intervention, as compared with diabetes support and education, on a) the use of medications as a binary outcome (odds ratios represent the risk of being on any medications of the given class by the end of follow-up), and b) the number of medications within each of three classes, at the start versus the end of the Look AHEAD trial period (incidence rate ratios represent the risk of increasing the medication number by one by the end of follow-up), in people who were and were not eligible for diabetes remission programs at baseline. ‘Net change/py’ represents the net change in medications in the given population, over the number of person-years of follow-up in that population – negative numbers are observed given that participants could stop medications. ILI = intensive lifestyle intervention; DSE = diabetes support and education; py = person-years.
